# Building genomes to understand biology

**DOI:** 10.1038/s41467-020-19753-2

**Published:** 2020-12-02

**Authors:** Alessandro L. V. Coradini, Cara B. Hull, Ian M. Ehrenreich

**Affiliations:** Molecular and Computational Biology Section, Department of Biological Sciences, University of Southern California, Los Angeles, CA 90089-2910 USA

**Keywords:** Synthetic biology, Genomics

## Abstract

Genetic manipulation is one of the central strategies that biologists use to investigate the molecular underpinnings of life and its diversity. Thus, advances in genetic manipulation usually lead to a deeper understanding of biological systems. During the last decade, the construction of chromosomes, known as synthetic genomics, has emerged as a novel approach to genetic manipulation. By facilitating complex modifications to chromosome content and structure, synthetic genomics opens new opportunities for studying biology through genetic manipulation. Here, we discuss different classes of genetic manipulation that are enabled by synthetic genomics, as well as biological problems they each can help solve.

## Abstract (web summary)

Advances in our ability to manipulate genetics leads to deeper understanding of biological systems. In this Perspective, the authors argue that synthetic genomics facilitates complex modifications that open up new areas of research.

## Introduction

**Fig. 1:**
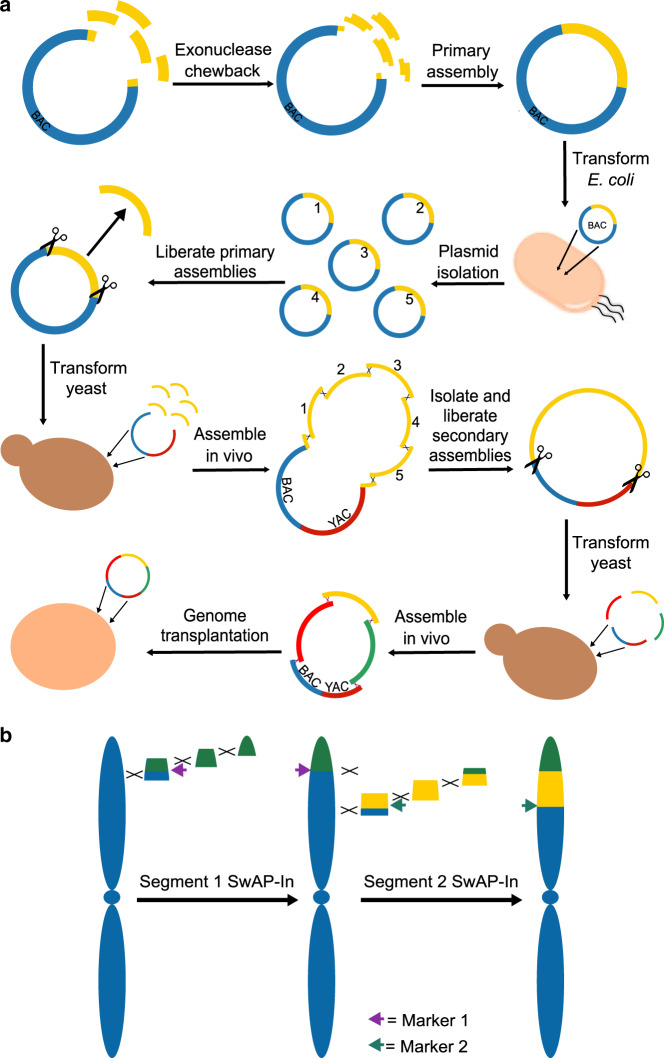
Methods for building chromosomes. **a** Synthetic chromosomes are constructed using a hierarchical assembly process^19^. This involves building primary assemblies from DNA fragments in vitro and then transforming these assemblies into *E. coli*. These primary assemblies can then be liberated from their plasmid backbones and co-transformed into yeast. Homologous recombination in yeast can then combine these primary assemblies to build a larger secondary assembly. If necessary, such secondary assemblies can be combined together in yeast to produce even larger molecules. In *Mycoplasma* (and potentially other organisms in the future), synthetic chromosomes can be activated through transplantation^9,20,132,133^. **b** In organisms that do not permit chromosome transplantation, an alternative strategy is to build chromosomes in vivo by iteratively replacing segments of the host cell’s genome. This figure is based on the SwAP-In approach described in yeast^10,22^.

Most biologists seek to understand life as it exists^1^. For many, this entails characterizing how genomes and molecular systems give rise to cellular life and its diversity^2^. The genetic manipulation of organisms has long played a critical role in this endeavor^3,4^, and innovations in methods for genetic manipulation continually expand the biological research that is possible^5–7^. In recent years, the synthesis of chromosomes, known as synthetic genomics, has emerged as a new form of genetic manipulation^8^. Megabase-sized chromosomes can now be generated from components synthesized de novo, obtained from naturally occurring genomes and other existing molecules, or a mixture of the two (Box 1, Fig. 1). During construction, the content and structure of these chromosomes can be modified relative to their natural templates to enable biological hypothesis testing^9,10^, as well as to make organisms more amenable to research and bioengineering^11^.

Because of their comparatively small size, viral chromosomes were the first to be synthesized. This began in the early 2000’s^12^ and a number of viral chromosomes have been constructed to date, including hepatitis C^13^, polio^14^, ΦX174^15^, a SARS-like coronavirus^16^, herpes type 1^17^, and the coronavirus that causes COVID-19^18^. However, by 2008, chromosome synthesis had progressed to cellular life forms, beginning with the human pathogenic bacterium *Mycoplasma genitalium*^19^. Subsequently, complete chromosomes were constructed for a number of other bacteria, including *Mycoplasma mycoides*^9,19,20^, *Caulobacter crescentus*^21^, and *Escherichia coli*^11^, as well as for the budding yeast *Saccharomyces cerevisiae*^10,22–26^. In some cases, these chromosomes were re-engineered in remarkable ways, such as through the complete elimination of nonessential genes^9^ or particular codons^11^, providing striking examples of the large-scale genetic manipulations that are enabled by synthetic genomics.

These foundational studies illustrate the tremendous advances that have occurred in synthetic genomics in roughly a single decade. They also suggest that moving forward, continued progress in synthetic genomics will facilitate increasingly complex genetic manipulations across a broader range of cellular organisms^27–29^. Here, we assume such progress and discuss different classes of genome-wide genetic manipulation that either are presently possible or will likely become feasible in the future. We focus on six specific classes of genetic manipulation enabled by synthetic genomics: restructuring, recoding, minimization, chimerism, organelle reengineering, and genome resurrection. For each class of genome-wide genetic manipulation, we explore fundamental biological questions that they can be used to address. Our goal in this perspective is to inspire scientists to utilize synthetic genomics to advance understanding of biological systems.

Box 1 Methods for building and activating chromosomes *Assembly of large DNA molecules*: Chromosomes are synthesized through the hierarchical assembly of smaller DNA molecules into progressively larger ones. This process begins with molecules that are hundreds of base pairs in length (“fragments”), which can be readily ordered from commercial vendors and are reviewed elsewhere^134,135^. Fragments are typically combined together and cloned into bacterial plasmids by Gibson^113,136,137^ or Golden Gate^138,139^ assembly. These are highly specific and scarless in vitro techniques that rely on annealing or restriction digestion and ligation of partially overlapping fragments^140^, respectively (Fig. 1a). Assemblies generated with these techniques are usually on a scale of 5–10 kb but can be as large as hundreds of kilobases^113,136–139^. However, larger in vitro assemblies are susceptible to shearing during handling^141^ and may not be tolerated by *E. coli*, which is used to store and replicate these molecules^19^. Thus, often smaller in vitro assemblies are constructed and then combined together into larger molecules using yeast, which is highly recombinogenic^19^. In yeast, multiple smaller assemblies can be co-transformed and combined through homologous recombination into a larger assembly contained within a single centromeric plasmid^19,44,45,142–144^ (Fig. 1a). This approach has been used to produce molecules greater than a megabase in size^9,19–21^. In the future, cell-free DNA replication systems may make it possible to bypass host organisms, such as *E. coli* and yeast, and to construct chromosome-sized molecules entirely in vitro^145,146^.

*Bringing synthetic chromosomes to life*: Chromosomes constructed in yeast must be transplanted into living cells. This involves transforming these molecules into suitable recipient cells and selecting for their retention, resulting in the complete replacement of a cell’s original chromosome^132^. To date, such chromosome transplantation has only been demonstrated in *Mycoplasma*, a bacterial genus that possesses the smallest known genomes of all independently replicating organisms^9,20,132,133^. Thus, for most species, synthetic chromosomes must instead be built through an iterative process in which existing genomic segments are successively replaced with synthetic assemblies using homologous recombination and selection. Several examples of this strategy have been reported, including switching auxotrophies progressively for integration^22^ in the yeast Sc2.0 project^10,22–26^ (Fig. 1b); stepwise integration of rolling circle amplified segments in *Salmonella typhimurium*^71^; conjugative assembly genome engineering (CAGE) in *E. coli*^69^; and replicon excision for enhanced genome engineering through programmed recombination in *E. coli*^11,70^. Over multiple cycles, these iterative, in vivo approaches can be used to produce entirely synthetic chromosomes that are biologically active.

## Genetic manipulations enabled by synthetic genomics

### Restructuring

Genome structure is thought to play an important role in evolution and phenotype^30,31^. Supporting this possibility, genome structure varies significantly across species^32^, and changes within species are also prevalent, often impacting traits^33^. While sequencing has led to the detection of many chromosome structural differences within and between species, rarely is it possible to know their biological significance through purely bioinformatic approaches^32^. Synthetic genomics enables the intentional restructuring of chromosomes, thereby facilitating research aimed at empirically determining the functional and phenotypic consequences of changes in genome structure. By restructuring chromosomes at different scales, scientists can directly probe the relationship between the layout of a genome and the features of an organism^32,34–40^.

**Fig. 2:**
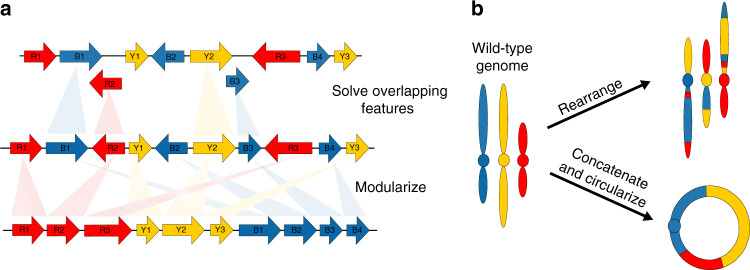
Restructuring. **a** Synthetic genomics makes it possible to engineer diverse chromosome structures. At a micro-scale, the arrangement of genetic material within genomic regions can be altered. For example, structural overlaps between adjacent genes can be eliminated (i.e., refactoring) or genes with related functions can be positioned next to each other (i.e., modularization). Such modifications can be implemented chromosome-wide. **b** On a macro scale, this includes the introduction of gross chromosomal rearrangements, such as deletions, insertion, inversions, and translocations, as well as the modification of chromosome number and conformation.

At the finest scale, the structures of genes and other functional elements can be altered. For example, many genes overlap another gene on the opposing DNA strand. Because of these overlaps, a genetic change in a gene on one strand might also affect the sequence of a gene on the other strand. Work to remove these overlaps from a large portion of the phage T7 genome showed that the elimination of such overlaps often has little to no phenotypic consequence^41^. This form of restructuring, which has been called refactoring^41^ (Fig. 2a), makes it possible to not only study the biological impacts of local genome structure but also to produce organisms that are more amenable to research. For example, refactoring may be necessary if one wants to globally replace certain codons with alternative codons^11^. However, it also bears mention that generating new overlaps between genes may be desirable in some cases, such as to limit the evolutionary potential of synthetic organisms^42^.

At an intermediate scale, restructuring can be used to rearrange genetic material within a genome. For example, in one-eighth of a synthetic *M. mycoides* genome, genes were reordered into modules based on common function^9^. This restructured genome produced a viable organism, raising questions about whether gene arrangement along a chromosome is functionally important and suggesting that someday it may be possible to generate organisms with completely modular genomes (Fig. 2a). However, similar work in other organisms, including multicellular species, is needed to more generally assess the functional and phenotypic consequences of gene arrangement. In these organisms, gene arrangement might have greater biological significance, for example by affecting the three-dimensional conformation of the genome in the nucleus.

Lastly, at the macro scale, restructuring can be used to modify how entire genomes are packaged into chromosomes (Fig. 2b). Strategies for chromosome restructuring inside living cells have been reported using CRISPR/Cas9 or restriction enzymes^32,34–39,43^. Most applications of these approaches sought to introduce one or a small number of targeted or random changes in genome structure. However, in budding yeast, these approaches were used to more extensively modify the organism’s genome structure by concatenating the 16 nuclear chromosomes into as few as one or two molecules^32,38^. Subsequently, it was shown that yeast, which naturally possesses linear chromosomes, will even tolerate a genome restructured into a single, circular chromosome^39^. These changes in genome structure had little impact on gene expression, but affected growth across environments, reproductive ability, and other cellular features. This suggests that the phenotypic impacts of restructuring may be mediated through non-transcriptional mechanisms.

Synthetic genomics enables even more extensive restructuring than what was described in the preceding paragraphs. For example, a genome could be simultaneously restructured at multiple of the aforementioned scales. This is a key part of the Sc2.0 project, in which loxP sites have been added between each pair of genes on a given synthetic chromosome to allow for random Cre-mediated site-specific recombination^10,22–26^. Because of these loxP sites, synthetic chromosomes constructed as a part of Sc2.0 facilitate the generation of large libraries of cells with diverse chromosome or genome structures through the induction of synthetic chromosome recombination and modification by LoxP-mediated evolution^44–51^. Analysis of cells produced by SCRaMbLE can reveal how changes in genome structure influence particular phenotypes or can be used to screen for genome structures that confer traits of interest. While SCRaMbLE is powerful and highly scalable, it also has some constraints, such as a need for de novo sequencing to determine the exact structures of output genomes and a likelihood that output genomes will vary in their gene content.

Moving forward, it will be desirable to generate similar genome restructuring libraries in other species. Such work may help clarify the role of genome restructuring in evolutionary change and other biological phenomena, such as the emergence of cancer. On the latter topic, genome restructuring events are observed in many cancers^52^. In some cases, it is even thought that massive, one-step genome restructuring events, known as chromothripsis, can drive oncogenic transformations^53^. Yet, our understanding of the relationship between cancer and genome restructuring is limited to what has been observed through genome sequencing. Synthetic genomic approaches may enable the controlled experimental characterization of the role of genome restructuring in cancer. Genome restructuring experiments also provide numerous other opportunities for biological discovery. For example, they may provide insights into how genome structure relates to three-dimensional genome conformation, the regulation of transcription, and the expression of phenotype in multicellular eukaryotes, similar to recent efforts to explore these questions in yeast^40^. Research along these lines could improve our understanding of development, thereby enhancing efforts to reprogram and differentiate cells in a controlled manner^54^.

### Recoding

**Fig. 3:**
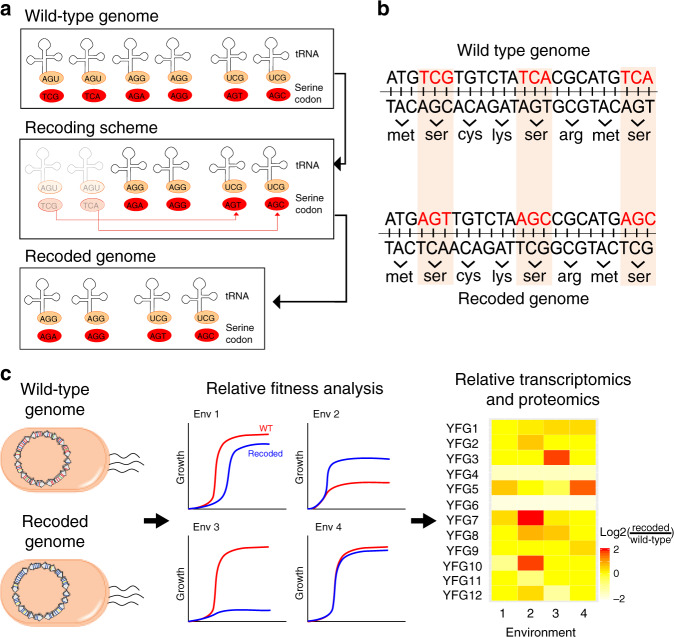
Recoding. **a**, **b** Certain codons can be replaced with alternative codons on a genome-wide scale^11,66,70^. Typically, such recoding involves substituting a codon with a synonymous codon, followed by the deletion of the corresponding tRNA for the original codon. Here, the number of synonymous codons for the serine amino acid is reduced from six to four by the genome-wide substitution of TCG and TCA codons by the synonyms AGT and AGC, respectively. This makes it possible for the codon and tRNA to be repurposed, for example, to incorporate nonnatural amino acids into proteins. However, in the future, recoding efforts may also involve non-synonymous codon substitutions. **c** Genome-wide recoding can be used to explore how codon usage affects the transcriptome, proteome, and fitness.

Codon usage is another feature of genomes that varies among species^55–57^. Differences in codon usage between genes in the same genome or between different genomes can affect transcription, translation, and other molecular processes within an organism^55,58,59^, providing a potential substrate for natural selection^60–62^. The importance of codon usage can be explored experimentally using recoding—the genome-wide replacement of particular codons with their synonyms (Fig. 3a, b). Recoding can be used to address questions about the molecular and evolutionary impacts of changes in codon usage^63,64^, and to produce organisms poised for biotechnology applications, such as the generation of organisms that utilize non-natural amino acids^65–67^ (Fig. 3c).

Both genome editing and synthetic genomics approaches enable recoding on a genome-wide scale^11,64,65,68–71^. Genome-wide manipulation of codon usage was first performed in *E. coli* with the objective of generating a strain in which all 314 TAG stop codons were converted to TAA^72^. Doing this required combining a genome editing strategy—multiplexed automated genome editing (MAGE)^68^—with a conjugation-based assembly method—CAGE^69^. MAGE produces site-specific nucleotide changes by utilizing short oligonucleotides and the λ phage β protein^68^ to catalyze homologous recombination between single-stranded DNA and a chromosome^73^. Although highly effective, MAGE is only capable of introducing a limited number of edits at a time. Thus, different regions of the genome were edited in parallel in distinct strains and then combined together using CAGE to produce strains containing as many as 80 synonymous codon changes^72^. This work highlights limitations to genome recoding through genome editing, including the number of codons that can be simultaneously modified, challenges in completely eliminating particular codons, and the need for sequence complementarity between editing reagents and a genome.

In contrast to genome editing-based recoding strategies, synthetic genomics is constrained only by what an organism will tolerate^11,70,71,74^. The most extensive, synthetic genomic-based recoding efforts thus far also occurred in *E. coli*. In one study, a 57 codon genome lacking the TAG stop codon, two arginine codons, two leucine codons, and two serine codons was designed and constructed as a series of ~50 kb assemblies^74^. These segments were assayed for viability using complementation tests but have yet to be assembled into a recoded genome. In a second study, a strategy for genome-wide recoding using direct replacement of *E. coli* genomic segments with DNA assemblies was developed^70^. This approach was recently applied genome-wide to recode certain serine codons from TCG to AGC and TCA to AGT, as well as to replace TAG stop codons with TAA^11^. The *E. coli* Syn61 strain produced by this work contains 18,214 codon modifications in total but shows little phenotypic change relative to its progenitor, illustrating the malleability of the genetic landscape. This work also demonstrated the potential to obtain new insight into functional differences between synonymous codons through the identification of idiosyncratic codons that do not tolerate recoding^70,74^.

The above projects show that genome-wide recoding is now feasible, at least in certain organisms. Based on these advances, a number of fundamental questions can now be explored using recoding. For example, to what extent can the genetic code be reduced? At the extreme, one could imagine trying to produce an organism that utilizes only 21 codons, one for each of the 20 amino acids and one for the stop. The generation of organisms with simplified genetic codes may make it possible to explore the consequences of tRNA utilization for fitness and evolvability across environments. Recoding may also make it possible to generate particular nonsynonymous changes globally. Such work could clarify the exchangeability (or lack thereof) of different amino acids and can reveal mechanisms by which the translation apparatus recovers from problems in translation^75^. Recoding may even shed light on ecological interactions between organisms or between living species and viruses^29^. Indeed, evidence suggests that such interactions can shape patterns of codon usage, likely due to shared environmental resources and selection upon translation^76^.

### Minimization

**Fig. 4:**
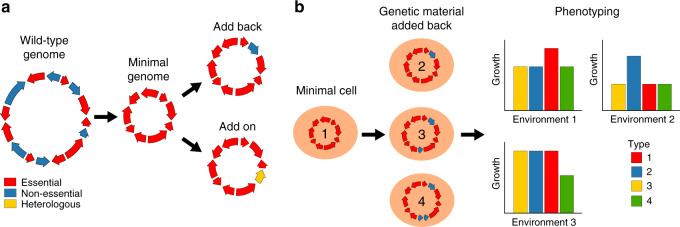
Minimization. **a** Minimal genomes lack non-essential genes^9^. After a minimal genome has been generated, non-essential genes can be added back to explore how they impact function and phenotypic outcomes. In addition, heterologous genes (add on) can be inserted to create new cell phenotypes. **b** Genetic material added back to minimal genomes may help elucidate how certain genes contribute to phenotype across environments.

A fundamental question in biology regards the minimal set of genes and physiological functions needed to support cellular life^77,78^. Synthetic genomics has made it possible to experimentally answer this question through genome minimization, the elimination of all non-essential genes from an organism’s genome^9^ (Fig. 4a). The output of genome minimization is an organism that only carries a core set of genes needed to sustain life and reproduction in a given environment. In the first and only genome minimization to date, a minimal *M. mycoides* genome was produced. *Mycoplasma* species have small genomes, with the smallest known genome of a cellular life form being that of *M. genitalium* (580 kb containing around 525 genes). Naturally occurring *M. mycoides* has a roughly one Mb genome containing nearly 1,000 genes. However, using multiple rounds of global transposon mutagenesis and a genome design-build-test cycle to eliminate non-essential genes, researchers produced a 531 kb synthetic *M. mycoides* genome with only 473 genes^9^. This minimal organism required genes involved in the expression of genomic information (i.e., transcription and translation), the cell membrane, cytoplasmic metabolism, and preservation of genome information, though 149 genes had unclear functions.

Mycoplasmas are extracellular parasites or commensals that have streamlined genomes adapted to life within a host^79^ and may have a single minimal genome state. In contrast, most unicellular organisms used in biological research, including *E. coli* and *S. cerevisiae*, have larger numbers of genes, more complicated genomes, and a greater tolerance for diverse environments^80,81^. For organisms like these, whether one or multiple potential minimal genomes exists, as well as how these minimal gene sets might depend on the environment, is not entirely clear. Because these organisms possess diverse metabolic modes and an abundance of functional redundancies at the genetic level^82^, there may be a potential for multiple minimal genomic states in a given condition. This question should be directly addressable in the future through synthetic genomic-based genome minimization efforts.

Minimization also has the potential to provide insights into multicellular organisms by improving understanding of how a single genome encodes diverse cell types. Minimization could be used to identify genes that are essential throughout all cell types, as well as genes that are essential for only particular cell types. By minimizing the genomes of organoids instead of cells, the essential gene set for cell–cell interactions that produce a given tissue might even be identified. However, a challenge with multicellular species, and potentially some unicellular species as well, will be alternative splicing, which can significantly impact the enzymatic activities, protein–protein interactions, and other functional attributes of proteins^83^. One could imagine performing minimization in a way that eliminates certain isoforms through the removal of only some exons or introns of a gene; however, doing this in a scalable manner will likely require technical innovation.

Across species, minimization also has the potential to help characterize the essential non-coding portion of genomes. In multicellular organisms, most transcripts are noncoding and many of these molecules have unknown biological significance^84,85^. Minimization of the noncoding portion of the genome could elucidate which of these transcripts are essential for life, which can serve as a starting point for determining their biological functions and mechanisms of actions. Similar logic can be employed for mobile genetic elements, which comprise most of the human genome and are abundant throughout the genomes of many other organisms as well^86,87^. Production of genomes in which such mobile elements have been removed could also have applied benefits, such as the production of crops that grow faster due to their reduced genome sizes or cell lines with enhanced genomic stability.

Minimization can also provide a foundation for studying the non-essential portions of genomes. Minimal genomes can serve as chassis to which nonessential genetic material can be added back or added on. We use these terms to refer to the analysis of nonessential genes or other DNA elements from the same or different organisms, respectively, through their addiction to a minimal genome (Fig. 4a, b). Such work may address how particular nonessential genes and pathways influence features of an organism, including fitness in a focal environment, tolerance to different environments, mutational robustness, and evolvability^88–90^. In multicellular organisms, add back of cell type-specific essential genes be useful for studying the roles of nonessential genes in enabling a core genome to produce a diversity of cell types. Add on genetics could also possibly be used to probe questions about how the addition of genetic material facilitates the evolution of new traits and species.

### Chimerism

**Fig. 5:**
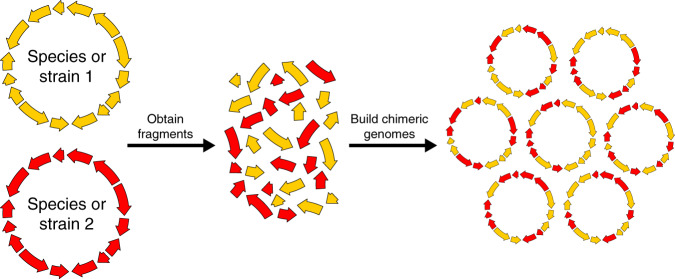
Chimerism. Synthetic genomic approaches can enable the generation of chimeric chromosomes that contain mosaics of genetic material from two or more strains or species. By generating and screening libraries of chimeric chromosomes, it may be possible to determine the genetic basis of traits that differentiate reproductively isolated organisms.

Chimerism is the combining of genetic material from multiple strains or species into a single organism (Fig. 5). This is standard practice in biology and bioengineering; for example, heterologous expression of pathways is often used to produce drugs and other valuable compounds^91–94^. However, synthetic genomics makes it possible to consider new forms of chimerism either through the programmed combination of specific genomic segments from different organisms, as has been shown in *E. coli*^95^, or random approaches, as has been shown with the application of SCRaMbLE to yeast interspecies hybrids^48^. However, present limitations in the generation of chimeras are the inabilities to easily hybridize more than a small number of loci^95^ and to conduct SCRaMbLE between chromosomes from different species^48^.

In the future, building and phenotyping libraries of genomes that are mosaics between two or more strains or species may enable the identification of functional genetic differences in situations that do not permit standard genetic mapping approaches, such as linkage and association mapping^96,97^ (Fig. 5). This might allow for mapping of loci that contribute to genetic incompatibilities or traits of interest between individuals or species that are not amenable to controlled crossing because hybrids are inviable or produce defective offspring. The generation of chimeras might also allow geneticists to map traits across larger evolutionary distances than can typically be experimentally explored by crosses, such as at the genus level or higher. Using chimerism for genetic mapping will require approaches for making recombinant chromosome libraries with the same genome structure but a high degree of genetic shuffling, as would typically be obtained from meiosis.

The production of chimeric genomes may also be of value in understanding the functional and phenotypic significance of species’ pan-genomes. Work in prokaryotes^98–100^, as well as both unicellular and multicellular eukaryotes^101,102^, has shown that individuals within species commonly harbor extensive differences in gene content. This gene content variation clearly plays a major role in many heritable phenotypes, but often it can be difficult to connect specific gene content differences to particular traits. For example, any two *E. coli* isolates may only share the minority of their genes^103–105^. Producing libraries of chimeric *E. coli* genomes (Fig. 5) could make it possible to examine how this substantial variation in gene content influences ecologically and clinically relevant traits, including antibiotic resistance, tolerance for different environmental conditions, and pathogenicity. It may also enable the identification of genetic interactions involving genes in the pan-genome, thereby shedding light on their functions^82^.

A probable limitation of chimerism will be transcriptional dysregulation resulting from the combining of chromosome segments from genetically diverged organisms. To overcome such issues, it may help to generate chimeras in which the protein-coding portions of the genomes vary while the noncoding portions remain the same. This could enable examination of how the proteome alone differs in function between contributing genomes. Of course, understanding how transcriptional regulation evolves within and between species is an important question as well. To address this, one could envision keeping the protein-coding portion of the genome the same but varying the noncoding portion. These ideas speak to how chimerism can be employed in sophisticated ways that go beyond crudely combining together DNA segments from different sources.

### Organelle reengineering

Nearly all eukaryotes possess mitochondria, with photosynthetic eukaryotes also harboring chloroplasts. These organelles arose through the internalization and co-option of bacteria^106,107^. Since their origins, organelles have lost the majority of their genes through mutational degeneration or translocation to the nuclear genome^108^. This means that only a small fraction of the essential genes for a given organelle are encoded within the organelle itself. Additionally, ongoing molecular evolution has resulted in substantial variability in organellar genome size and content across species. For example, mitochondrial genomes range from 6 kb in the malaria parasite *Plasmodium falciparum* to 11 Mb in the plant *Silene conica*^109^. Although the origins and ongoing evolution of these organelles can be studied using sequencing and bioinformatics, such approaches are limited in their abilities to explore the early stages of organelle evolution and do not permit mechanistic hypothesis testing by experimentation^109,110^. In addition, techniques for genetically manipulating organellar genomes are less developed than methods for modifying the nuclear genome, though methods for directing specific metabolic enzymes^111^ or base editors to the mitochondria have been described^112^.

**Fig. 6:**
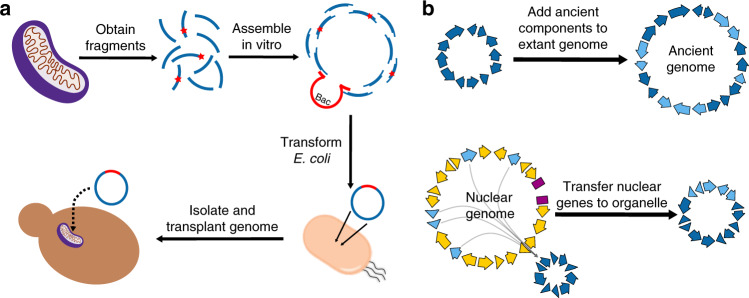
Organelle reengineering. **a** Organellar genomes can be constructed by harvesting and modifying natural genetic material or by performing de novo synthesis^113^. **b** Synthesis of organellar genomes can be used to study a number of problems, including, but not limited to, the early evolutionary stages following endosymbiosis and cytonuclear genetic interactions. Note: the purple rectangles represent telomeres.

To help address gaps in our understanding of evolution and genetics, organellar genomes can be synthesized^113^ (Fig. 6a). Relative to nuclear chromosomes, organellar genomes are easier to synthesize because they are typically smaller and contain fewer genes. Once constructed, these synthetic organellar genomes can then be transformed into organelles within cells using biolistic approaches^114^, though higher throughput transplantation methods are likely to emerge in the future^28^. By constructing likely evolutionary intermediates and activating them in organisms, it may be possible to reproduce key steps in organellar evolution (Fig. 6b). Furthermore, transferring genes back from the nuclear genome to the mitochondrial genome may further clarify the benefits of encoding these genes in the nucleus^115,116^ (Fig. 6b). Because genetic variants in the organellar genomes frequently contribute to incompatibilities between strains and species, such work can also provide insight into the role of organellar evolution on reproductive isolation^115^. Beyond these evolutionary questions, synthetic genomics might simply aid in mapping organellar mutations and genetic variants with phenotypic effects. For example, to date, more than 250 point mutations in the human mitochondrial genome have been implicated with roles in disease^117^. The human mitochondrial genome is only ~17 kb, suggesting that it should be possible to build and transplant comprehensive variant libraries. Such work could further clarify the contribution of the mitochondria to disease and other traits.

### Genome resurrection

Further in the future, another biological application of synthetic genomics may be the synthesis and activation of extinct organisms’ genomes. We refer to this as a genome resurrection due to its similarity to the resurrection and study of ancient biomolecules^118–120^. Extinct genomes sequences may be determined by sequencing ancient DNA, bioinformatic inference from extant genomes, or a combination of the two^121^. Synthesis of all or part of extinct genomes can provide substrates for the broader analysis of the molecular evolution of gene and pathway functions^118–120^. This is true regardless of whether these genomes are activated to produce living organisms. In cases in which it is appropriate, activation of these genomes can be used in de-extinction^122–126^. Presently, using genome editing to make extant species similar to their extinct relatives is being discussed^122,123^. Yet, the number of edits needed to convert one species into another could prove prohibitive, requiring the use of synthetic genomics in the future. There may be benefits to de-extinction in some cases, though careful ethical consideration will be imperative^124–126^.

Genome resurrection could also potentially enable research aimed at understanding key steps in molecular and phenotypic evolution. For example, it could help elucidate the origins and diversification of eukaryotes. The favored theory for the origin of eukaryotes is that the ancestral eukaryotic cell was produced through the fusion of an archaeon and a bacterium^127^. The recent isolation of a species of Asgard archaea, which are the closest extant relatives of the archaeon that gave rise to eukaryotes, specifically suggests eukaryotes evolved due to their archaeal ancestor engulfing and endogenizing a syntrophic bacterium^128^. Utilizing this information, it might be possible to construct and activate a genome resembling the earliest eukaryote. This would provide a model system for studying the initial evolution of eukaryotes, as well as for examining how additional defining eukaryotic features subsequently evolved. For example, what were the origins of the nucleus, cytoskeleton, and specialized organelles^129^? The addition of genetic material to the ancestral eukaryote model could be used to study the mechanisms underlying these intermediate steps in eukaryotic evolution. Similar logic could be applied to later eukaryotic innovations, such as multicellularity and potentially even the nervous system.

## Conclusion

During the last decade, synthetic genomics has gone from science fiction to reality, giving rise to fundamentally new ways to genetically manipulate biological systems. Relative to more conventional approaches for genetic manipulation, synthetic genomics is distinguished by both the large numbers and diversity of genetic changes that can be introduced. Indeed, as discussed throughout this paper, for certain single-celled organisms, it is now possible to entirely modify genome structure and content. In the future, technological advances should enable similar manipulations in a much broader array of species, including multicellular organisms. As we have attempted to emphasize, the ability to perturb biological systems in such a manner has the potential to provide deep insights into a wide range of fundamental topics in biology that remain only partially understood. Arguably, the overarching theme connecting all of the biological disciplines, classes of genome-wide genetic manipulation, and specific questions that we have discussed are that synthetic genomics will likely play a key role in achieving a deep mechanistic understanding of how biological systems work and came to be.

Of course, as synthetic genomics progresses, ethical considerations will be a major concern. Already with efforts to date, questions have been raised about the acceptable bounds of genetic modification in existing life forms^130,131^. The degree of manipulation that is now possible with synthetic genomics challenges our notions of species, as many of these genetic manipulations that we have described will produce organisms that significantly differ at the sequence level and may be reproductively isolated from their progenitors. Is it acceptable for people to generate such organisms? We believe that such manipulations are necessary to advance biology as a field, though valid questions exist about such manipulations. For example, would such manipulations be allowed in vertebrates or human pathogens? A more practical ethical concern is the escape of organisms whose genomes have been heavily manipulated into the environment. However, as others have argued, ethical, governance, and containment policies, such as proposed for Sc2.0^131^, as well as modification of synthetic genomes to make them unfit outside the lab, can help alleviate many ethical concerns.

In conclusion, synthetic genomics represents the genesis of a new era of biology in which scientists will increasingly transition from simply reading genomes to writing them^28,29^. Many fundamental questions about biological systems that historically could only be addessed through inferential, bioinformatic analyses may now or will soon be amenable to experimentation using synthetic genomics strategies. It is hard to imagine that modifying biological systems through synthetic genomic approaches will not produce new, previously inaccessible insights. This speaks to how building genomes can be used to determine the design rules that underpin life. We expect that work along these lines will not only enhance our understanding of biology, but it will help maximize the potential for genetically programming cellular organisms for human benefit in the future.
